# A Novel Y-Shaped Pegylated Recombinant Human Growth Hormone for Children With Growth Hormone Deficiency

**DOI:** 10.1210/clinem/dgae816

**Published:** 2024-11-28

**Authors:** Yan Liang, Haiyan Wei, Fan Yang, Hua Zhang, Linqi Chen, Hui Yao, Xiaoming Luo, Xinran Cheng, Yu Yang, Qun Lian, Hongwei Du, Tang Li, Pin Li, Gaixiu Zhang, Fuying Song, Liyang Liang, Deyun Liu, Shunye Zhu, Haihong Gong, Chunxiu Gong, Xiangao Cheng, Zhuangjian Xu, Yaping Ma, Zhe Su, Weidong Zhou, Ruoyi He, Yalin Yin, Li Sun, Xiaoping Luo

**Affiliations:** Department of Pediatrics, Tongji Hospital, Tongji Medical College, Huazhong University of Science and Technology, Wuhan 430030, China; Department of Endocrinology, Genetics and Metabolism, Children's Hospital Affiliated to Zhengzhou University, Henan Children's Hospital, Zhengzhou Children's Hospital, Zhengzhou 450018, China; Department of Pediatrics, Key Laboratory of Birth Defects and Related Diseases of Women and Children (Sichuan University), Ministry of Education, West China Second University Hospital, Sichuan University, Chengdu 610066, China; Department of Pediatrics, Sanya Central Hospital, Sanya 572000, China; Department of Endocrinology, Metabolism and Genetic Disorders, Children's Hospital of Soochow University, Suzhou 215003, China; Department of Genetic Metabolism and Endocrinology, Wuhan Children's Hospital, Tongji Medical College, Huazhong University of Science and Technology, Wuhan 430015, China; Department of Pediatrics, Zhejiang Provincial People's Hospital, Hangzhou 310014, China; Department of Pediatric Endocrinology, Genetics, and Metabolism, The Affiliated Women's and Children's Hospital, School of Medicine, University of Electronic Science and Technology of China, Chengdu Women's and Children's Central Hospital, Chengdu 610031, China; Department of Endocrinology, Genetics and Metabolism, Jiangxi Provincial Children's Hospital, Nanchang 330006, China; Department of Pediatrics, The First Affiliated Hospital of Xiamen University, Xiamen 361003, China; Department of Pediatrics, The First Affiliated Hospital, Jilin University, Changchun 130031, China; Department of Pediatric Endocrinology and Metabolism, Women and Children's Hospital, Qingdao University, Qingdao 266035, China; Department of Endocrinology, Children's Hospital of Shanghai, Shanghai Jiao Tong University, Shanghai 200040, China; Department of Pediatric Endocrinology, Shanxi Provincial Children's Hospital, Taiyuan 030006, China; Department of Endocrinology, Children's Hospital, Capital Institute of Pediatrics, Beijing 100020, China; Department of Pediatric Endocrinology, Sun Yat-Sen Memorial Hospital, Sun Yat-Sen University, Guangzhou 510120, China; Department of Pediatrics, The Second Hospital of Anhui Medical University, Hefei 230601, China; Department of Pediatrics, The Third Affiliated Hospital, Sun Yat-sen University, Guangzhou 510630, China; Department of Pediatrics, The First Affiliated Hospital of Nanjing Medical University, Nanjing 210029, China; Department of Endocrinology, Beijing Children's Hospital, Capital Medical University, Beijing 100045, China; Department of Pediatrics, The First Affiliated Hospital of Anhui Medical University, Hefei 230031, China; Department of Pediatrics, Anhui Public Health Clinical Center, Hefei 230012, China; Department of Pediatrics, Wuxi Fourth People's Hospital, Wuxi 214101, China; Department of Pediatrics, Wuxi Fourth People's Hospital, Wuxi 214101, China; Department of Endocrinology, Shenzhen Children's Hospital, Shenzhen 518038, China; Office of General Manager, Xiamen Amoytop Biotech Co Ltd, Xiamen 361028, China; Office of General Manager, Xiamen Amoytop Biotech Co Ltd, Xiamen 361028, China; Department of Translational Medicine, Xiamen Amoytop Biotech Co Ltd, Xiamen 361028, China; Office of General Manager, Xiamen Amoytop Biotech Co Ltd, Xiamen 361028, China; Department of Pediatrics, Tongji Hospital, Tongji Medical College, Huazhong University of Science and Technology, Wuhan 430030, China

**Keywords:** Y-shape pegylated rhGH, growth hormone, pediatric, growth hormone deficiency, long-acting growth hormone

## Abstract

**Context:**

Pegpesen is a novel Y-shape pegylated recombinant human growth hormone (rhGH) for once-weekly treatment of children with growth hormone deficiency (GHD).

**Objective:**

This work aimed to evaluate the efficacy and safety of Pegpesen in children with GHD vs daily rhGH.

**Methods:**

A multicenter, randomized, controlled phase 3 clinical trial was conducted at 23 centers in China with a duration of 52 weeks’ treatment. There were 391 pediatric participants diagnosed with GHD. Participants were randomly assigned 2:1 to a weekly Pegpesen group (140 μg/kg/week) or a daily rhGH group (245 μg/kg/week) for 52 weeks. The primary end point was the growth velocity (GV) at 52 weeks, and the secondary end points mainly involved changes from baseline in height SD scores for chronological age and bone age (ΔHt SDS CA and ΔHt SDS BA).

**Results:**

At 52 weeks, the least squares mean (LS means) of GV was 9.910 cm/year in the Pegpesen group and 10.037 cm/year in the daily rhGH group. The LS means difference between groups was −0.127 (95% CI, −0.4868 to 0.2332), confirming that weekly Pegpesen is noninferior to daily rhGH. The LS means of ΔHt SDS CA, ΔHt SDS BA, were similar across both groups (all *P* > .05). Safety profiles and adherence were comparable.

**Conclusion:**

Pegpesen was noninferior to daily rhGH, with similar safety, lower dosage requirements, thus presenting a new therapeutic option for children with GHD.

Growth hormone deficiency (GHD) is a syndrome caused by various factors and is associated with alterations in growth, body composition, and metabolism, affecting approximately 1 in 5000 children (1). The effectiveness and safety of daily recombinant human growth hormone (rhGH) for treating GHD in children have been extensively proven (2-4). However, numerous studies have highlighted poor adherence or nonadherence to daily rhGH treatment, primarily due to the need for frequent injections, which cause distress for some children, thereby affecting the achievement of linear growth targets (5-9). In recent years, various long-acting growth hormone (LAGH) formulations with extended half-lives have been developed worldwide to reduce administration frequency, enhance patient compliance, and consequently improve clinical outcomes (10, 11). The development of LAGH formulations generally falls into 2 categories: manipulation of drug release from subcutaneous depots, such as microsphere formulations, and manipulation of in vivo clearance from circulation, such as polyethylene glycol (PEG) formulations (3). The latter represents a leading technology extensively applied to enhance the performance of biotech drugs (12). Compared to rhGH, PEG-modified LAGH exhibits enhanced stability within the bloodstream and increased protection against proteolytic degradation. It also has the potential to mitigate or entirely avert immunogenicity under specific conditions, and may contribute to a reduction in toxicity (13, 14). Presently, in China, only one PEGylated LAGH, PEG-rhGH, has received approval (15, 16). Nonetheless, its phase 3 trial was narrowly focused on evaluating efficacy and safety over a limited 26-week period, and the medication's high cost has limited its availability (17). Therefore, ongoing research and development efforts in PEGylated LAGH are crucial for enhancing treatments for children with GHD.

Pegpesen is composed of 40-kDa Y-shaped branched PEG chains conjugated to the alpha-amino group in the epsilon-amino group of lysine in the side chain of rhGH, resulting in a mean half-life of 65 to 120 hours, which is 20 times longer than that of daily rhGH (18). Distinguished from other LAGH products both domestically and internationally, Pegpesen features unique long-acting technology, biological activity, and pharmacokinetic characteristics in vivo (17-21). In comparison, the currently available PEG-rhGH on the market employs U-shaped branched *N*-terminal PEGylated rhGH (UPEG-rhGH) and has a mean half-life of 31 hours (18, 22). As demonstrated in our prior patent, Y-shaped branched PEGylated rhGH exhibited superior bioactivity compared to UPEG-rhGH. This indicates that the required dose of Pegpesen to achieve equivalent efficacy with daily rhGH might be lower than that of UPEG-rhGH, offering a promising avenue for optimized dosing strategies (18).

The phase 2 study of Pegpesen confirmed that its efficacy and safety at a dosage of 120 to 140 μg/kg/week were comparable to that of daily GH at 35 μg/kg/day (18). Furthermore, quantitative analysis of pharmacological data indicated that the change in the area under the curve of insulin-like growth factor-1 (IGF-1) from baseline (ΔIGF-1 AUC) was most similar between the Pegpesen 140 μg/kg/week group and the daily rhGH group at 12 weeks (specific data not disclosed), suggesting comparable efficacy between the two treatment regimens. Consequently, for the phase 3 study, the Pegpesen dosage was set at 140 μg/kg/week, a lower therapeutic dose compared to other LAGHs on the market (160-660 μg/kg/week) (17, 19-21), which may contribute to enhanced long-term safety in children with GHD.

This phase 3 clinical trial further confirmed the efficacy and safety of weekly Pegpesen (Xiamen Amoytop Biotech Co Ltd) at a dosage of 140 μg/kg/week compared to daily rhGH (Norditropin, Novo Nordisk) in children with GHD. These findings provide robust evidence supporting the clinical application of Pegpesen and expand the treatment options available for GHD.

## Materials and Methods

### Study Design

This was a multicenter, randomized, open-label, active-controlled phase 3 clinical trial (ClinicalTrials.gov: NCT04513171), conducted across 23 centers in various regions of China, including central, eastern, southern, western, and northern areas from May 26, 2020, to July 10, 2023.

Participants were randomly assigned at a 2:1 ratio to receive weekly subcutaneous injections of Pegpesen (140 μg/kg/week) or daily rhGH (245 μg/kg/week) for 52 weeks. Sex and GH peak concentration (stratified as ≤5 μg/L, <5 μg/L to ≤7 μg/L, and >7 μg/L) were considered as stratification factors. Efficacy, adverse events, safety-related laboratory tests, and treatment adherence were assessed at 4, 12, 24, 36, and 52 weeks, as well as 5 weeks after discontinuation of the drug.

The study protocol was approved by the institutional review board/ethics committee at all participating centers. All procedures were conducted in accordance with the International Council on Harmonization guidelines for Good Clinical Practice, the ethical principles outlined in the Declaration of Helsinki, and the regulatory standards of China. Prior to enrollment in the study, written informed consent was obtained from the parents and/or legal guardians of the children, with assent also obtained from the children themselves, where appropriate based on their age.

### Participants

Participants underwent an 8-week screening period before enrollment. Inclusion criteria were as follows: (1) confirmed diagnosis of GHD prior to screening, characterized by a height SD score (Ht SDS) of less than −2 based on the Chinese general population standard for age (23), growth velocity (GV) of 5.0 cm/year or less, GH peak level of less than 10 ng/mL induced by 2 different stimulants, and bone age (BA) lower than chronological age (CA) (girls younger than 10 years, boys younger than 11 years); (2) prepubertal children (girls younger than or equal to 10 years, boys younger than or equal to 11 years), with external genitalia at Tanner stage I and age older than 3 years; (3) uniformly short stature with normal intellectual development; (4) IGF-1 levels lower than the median level of IGF-1 in children of the same age and sex.

Exclusion criteria were (1) prior use of rhGH and sex hormone therapies; (2) history of or current presence of malignancy and/or intracranial tumor; (3) severe allergic tendencies; (4) other types of growth abnormalities, such as idiopathic short stature, Turner syndrome, thyroid hormone deficiency, hypoadrenalism, and antidiuretic hormone deficiency; (5) other medical conditions that may hinder growth, such as liver dysfunction, diabetes, malnutrition, and deformities; (6) Cobb angle greater than 15 degrees in individuals with scoliosis.

### Efficacy End Points

The primary efficacy end point was the annual GV (measured in cm/year) of children with GHD treated with weekly Pegpesen compared to daily rhGH after 52 weeks of therapy. GV was determined by linear regression of each child’s height against the exact date based on height measurements.

Secondary efficacy end points primarily included changes in height SD score for chronological age (ΔHt SDS CA), height SD score for bone age (ΔHt SDS BA), and growth velocity (ΔGV) from baseline to 52 weeks, as well as serum levels of IGF-1 and insulin-like growth factor-binding protein 3 (IGFBP-3) at 52 weeks of treatment. Serum IGF-1 and IGFBP-3 levels were measured at baseline, and before administration at weeks 4, 12, 24, 36, and 52 to track their continuous changes.

### Quantitative Pharmacological Model

A quantitative pharmacological model of Pegpesen was developed for children with GHD, using serum IGF-1 levels of participants from Pegpesen phase 2 and phase 3 trials as the pharmacodynamic (PD) index. The IGF-1 characteristics of children receiving Pegpesen at doses of 100 μg/kg/week, 120 μg/kg/week, and 140 μg/kg/week were simulated separately, and quantitative pharmacological models for Pegpesen at different dosages were established. Evaluation of IGF-1 SDS and ΔIGF-1 AUC levels aided in comprehensively assessing the efficacy and safety of different dosage groups, providing a basis for dosage selection.

### Safety Assessments

Safety assessment included monitoring adverse events, vital signs, physical examinations, laboratory tests, changes in imaging findings compared to baseline, evaluation of immunogenicity, and instances of early withdrawal. Immunogenicity of the study drug was assessed by detecting the presence of anti-GH antibodies in participants before and after treatment. Anti-GH antibodies for both Pegpesen and daily rhGH were detected using a validated enzyme-linked immunosorbent assay, and neutralizing antibodies were further confirmed with a cell proliferation assay. A polyclonal antihuman GH antibody (R&D Systems, catalog No. AF1067, RRID: AB_354573) was used as the positive control.

### Adherence

Calculation the treatment adherence of the Pegpesen group and daily rhGH group according to the following method: adherence = (doses administered/doses prescribed) × 100%.

### Statistical Analysis

This study employed a noninferiority design, setting a noninferiority margin of −1.8 cm/year. Participants were allocated to the Pegpesen group and the daily rhGH group at a ratio of 2:1. Considering an estimated 20% withdrawal and dropout rate and regulatory requirements, it was determined that 360 children would need to be enrolled.

The individuals who were randomly assigned and received at least one dose of treatment were included in the full analysis set. The safety analysis set contained all randomly assigned children who received at least one treatment. Missing data were imputed by the last observation-carried-forward method. Analysis of covariance was employed, considering baseline GV, sex (male and female), GH peak concentration (≤5 μg/L, <5 μg/L to ≤7 μg/L, >7 μg/L), study center as a covariate, and treatment group as an independent variable to compare between groups.

Statistical analyses were performed using SAS 9.4 (SAS Institute Inc). All statistical tests were 2-tailed. A *P* value of less than or equal to .05 was considered statistically significant (unless otherwise stated).

## Results

### Baseline Characteristics

A total of 757 individuals underwent screening for this study, among whom 391 participants were randomly assigned to receive either weekly Pegpesen (n = 261) or daily rhGH (n = 130). The primary reasons for screening failures were that 47% of the individuals exhibited IGF-1 levels higher than those typically observed in children of the same age and sex, while 43% failed to meet the diagnostic criteria for GHD. Both the full analysis set and safety analysis set populations comprised 391 children. In all, 96% of participants completed the study. The mean ages, GV, Ht SDS CA, Ht SDS BA, IGF-1 levels, and GH peak concentration distribution were largely similar in both treatment groups. Overall, demographic characteristics and other baseline parameters were well-balanced between the two groups (Table 1).

**Table 1.** Study demographics and baseline characteristics (full analysis set)

	Pegpesen group (N = 261)	Daily rhGH group (N = 130)	*P*
Mean chronological age, y (SD)	6.68 (2.11)	7.03 (2.20)	.138
No. of male cases (%)	172 (65.9%)	87 (66.9%)	.840
Mean height, cm (SD)	108.37 (11.57)	109.94 (11.80)	.231
Mean weight, kg (SD)	18.46 (4.92)	18.83 (4.75)	.325
Mean BMI (SD)	15.47 (1.65)	15.34 (1.35)	.790
Mean growth velocity, cm/y (SD)	3.53 (1.39)	3.49 (1.31)	.600
Mean Ht SDS CA (SD)	−2.55 (0.61)	−2.57 (0.64)	.891
Mean Ht SDS BA (SD)	4.53 (10.16)	4.68 (10.64)	.439
Mean IGF-1 level, ng/mL (SD)	104.26 (43.85)	107.14 (42.48)	.247
Mean IGF-1 SDS (SD)	−0.90 (0.65)	−0.95 (0.62)	.462
Mean IGFBP-3 level, ng/mL (SD)	3.66 (0.88)	3.58 (0.88)	.724
Mean GH peak concentration, μg/L (SD)	6.59 (2.24)	6.43 (2.29)	.584
GH peak concentration group, n (%)			.935
≤5 μg/L	60 (23.0%)	30 (23.1%)	
<5 μg/L to ≤7 μg/L	78 (29.9%)	41 (31.5%)	
>7 μg/L	123 (47.1%)	59 (45.4%)	

Abbreviations: BMI, body mass index; Ht SDS BA, height SD score for bone age; Ht SDS CA, height SD score for chronological age; IGF-1, insulin-like growth factor-1; IGFBP-3, IGF binding protein 3; rhGH, recombinant human growth hormone; SDS, SD score.

### Efficacy

#### Primary efficacy end points

The least square means (LS means) of GV after 52 weeks of treatment were 9.910 cm/year in the Pegpesen 140 μg/kg/week group and 10.037 cm/year in the daily rhGH 245 μg/kg/week group. The LS means difference between the two groups was −0.127 (95% CI, −0.4868 to 0.2332), indicating the noninferiority of Pegpesen compared to daily rhGH (Table 2).

**Table 2.** Efficacy end points (full analysis set)

	LS means	95% CIL	95% CIU
GV			
Pegpesen group (N = 261)*^a^*	9.910	9.6475	10.1721
daily rhGH group (N = 130)*^a^*	10.037	9.6973	10.3760
Pegpesen-daily rhGH group	−0.127	−0.4868	0.2332
ΔHt SDS CA			
Pegpesen group (N = 261)*^b^*	0.873	0.8247	0.9215
daily rhGH group (N = 130)*^b^*	0.902	0.8395	0.9647
Pegpesen-daily rhGH group	−0.029	−0.0955	0.0375
ΔHt SDS BA			
Pegpesen group (N = 261)*^c^*	−2.997	−3.8323	−2.1627
daily rhGH group (N = 130)*^d^*	−3.019	−4.0987	−1.9393
Pegpesen-daily rhGH group	0.022	−1.1250	1.1680
ΔGV			
Pegpesen group (N = 261)	6.394	6.1319	6.6565
daily rhGH group (N = 130)	6.521	6.1817	6.8604
Pegpesen-daily rhGH group	−0.127	−0.4868	0.2332

Abbreviations: CIL, lower bound of CI; CIU, upper bound of CI; GV, growth velocity; LS means, least squares mean; rhGH, recombinant human growth hormone; ΔGV, changes in growth velocity from baseline to 52 weeks; ΔHt SDS BA, changes in height SD score for bone age from baseline to 52 weeks; ΔHt SDS CA, changes in height SD score for chronological age from baseline to 52 weeks.

^*a*^An analysis of covariance model including group, baseline GV, sex, GH peak concentration, and center was used for analysis.

^*b*^ *P* less than .001 compared to baseline.

^*c*^ *P* equals .259 compared to baseline.

^*d*^ *P* equals .138 compared to baseline.

**Figure 1.**
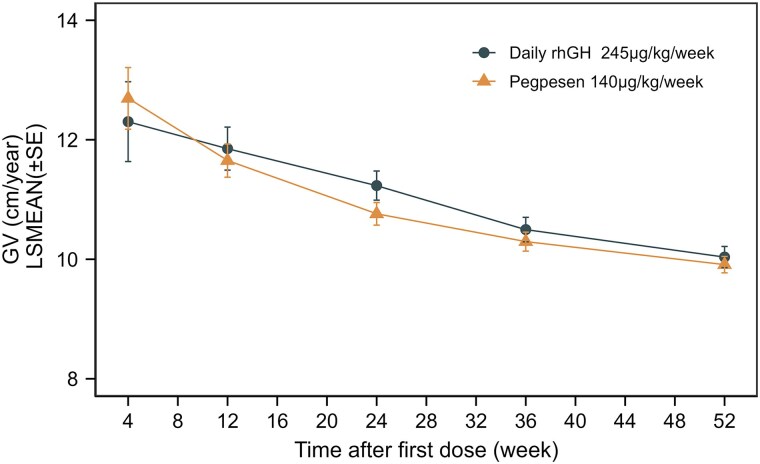
Annualized growth velocity (cm/year) by visit (full analysis set). Abbreviations: GV, growth velocity; LSMEAN, least squares mean; rhGH, recombinant human growth hormone.

The trend of average GV changes over the course of 12, 24, 36, and 52 weeks of treatment was consistent between the two groups (Fig. 1), demonstrating similar height improvement in both groups from baseline to 52 weeks.

During the 52-week treatment period, 8 participants (8/261) in the Pegpesen group and 1 participant (1/130) in the daily rhGH group entered puberty. Excluding data from these individuals, the GVs for the two groups were 9.947 cm/year and 10.063 cm/year, respectively. The LS means difference was −0.116 (95% CI, −0.4797 to 0.2476), consistent with the main analysis results and confirming that weekly Pegpesen is noninferior to daily rhGH.

#### Secondary efficacy end points

After 52 weeks of treatment, Ht SDS CA significantly improved both in the Pegpesen and daily rhGH groups compared to baseline (both *P* < .001), with the trends over the course of 4, 12, 24, 36, and 52 weeks being similar in the two groups (data not shown). Additionally, Ht SDS BA in both treatment groups exhibited a decreasing trend compared to baseline, without statistically significant changes (*P* = .259 and 0.138, respectively), suggesting that neither Pegpesen nor daily rhGH significantly accelerated BA growth or induced premature epiphyseal closure. The ΔGV significantly improved in both groups at 52 weeks (both *P* < .001 compared to baseline) and the LS means difference between groups supported the noninferiority observed in primary efficacy end points (see Table 2).

**Figure 2.**
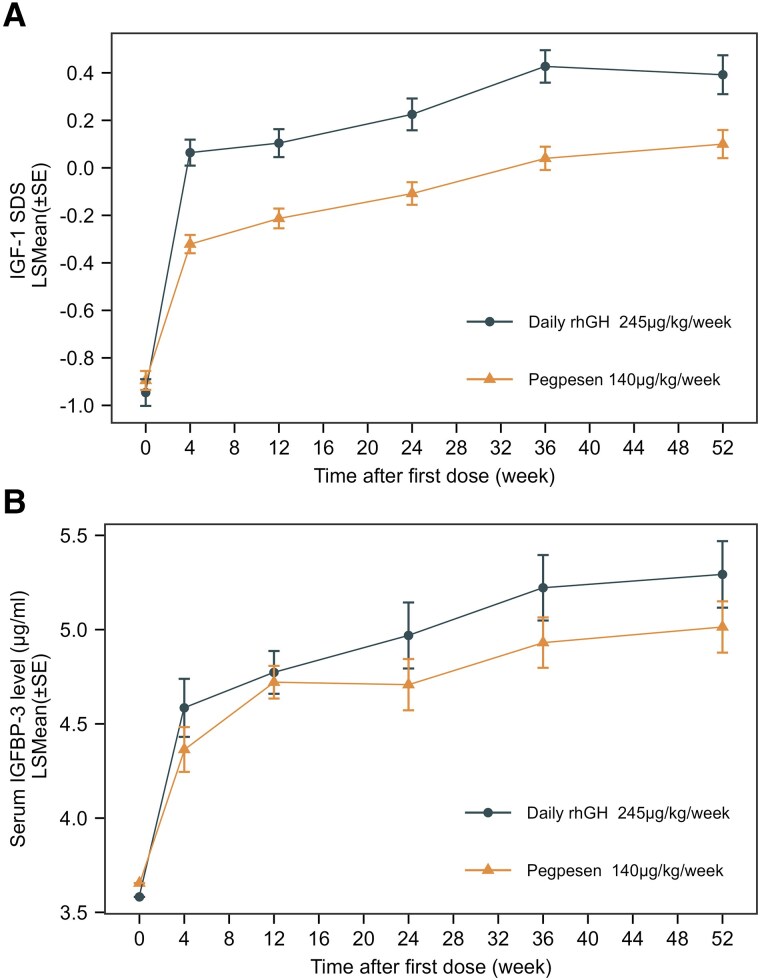
Observed IGF-1 SDS and IGFBP-3 profiles following Pegpesen and daily rhGH administration. A, At 52 weeks of treatment, IGF-1 SDS levels significantly improved both in the Pegpesen and daily rhGH groups compared to baseline (both *P* < .001). *P* = .0041 for intergroup comparisons. B, IGFBP-3 levels also significantly improved compared to baseline both in the Pegpesen and daily rhGH groups (both *P* < .001). *P* = .004 for intergroup comparisons. The statistical difference between the 2 groups was mainly due to different frequency of administration, thus the test results represented trough concentration in the Pegpesen group and peak concentration in the daily rhGH group. Abbreviations: IGF-1, insulin-like growth factor-1; IGFBP-3, insulin-like growth factor-binding protein 3; LSMEAN, least squares mean; rhGH, recombinant human growth hormone; SDS, SD score.

Furthermore, at 52 weeks of treatment, the levels of IGF-1 SDS and IGFBP-3 significantly increased in both groups compared to baseline (*P* = .041 and *P* = .004 for intergroup comparisons, respectively) (Fig. 2). However, the statistical difference between the two groups was primarily due to the weekly administration of Pegpesen vs daily administration of rhGH. As a result, the regimen design's IGF-1 and IGFBP-3 sampling points (preadministration testing) correspond to the trough concentration for the Pegpesen group and the peak concentration for the daily rhGH group, thus direct comparability was not feasible.

### Quantitative Pharmacological Model

Individual pharmacokinetic/pharmacodynamic (PK/PD) parameters of 292 individuals with GHD treated with Pegpesen were collected from both this study and a previous phase 2 trial. The IGF-1 exposures at 12 and 52 weeks were comparable, indicating achievement of steady state before 12 weeks, thus resulting in similar ΔIGF-1 AUC values at 12 and 52 weeks. Furthermore, the ratio of ΔIGF-1 AUC after the first dose to that after the last dose was 1.06 to 1.21, suggesting negligible accumulation of Pegpesen following multiple doses (Table 3).

**Table 3.** Levels of ΔIGF-1 area under the curve in different dose groups of Pegpesen

Dose, μg/kg	Wk	ΔC_max_, ng/mL	ΔIGF-1 AUC, ng × h/mL
100	0	95.7 (37.3)	9190 (41.7)
100	12	108 (36.1)	11700 (40.8)
100	52	108 (36.1)	11800 (40.8)
120	0	107 (35.9)	10700 (39.8)
120	12	121 (34.6)	14000 (38.8)
120	52	121 (34.6)	14100 (38.8)
140	0	117 (34.9)	12100 (38.3)
140	12	133 (33.5)	16200 (37.1)
140	52	133 (33.5)	16300 (37.1)

Abbreviations: ΔC_max_, change in maximum concentration from baseline; ΔIGF-1 AUC, change in the area under the curve of insulin-like growth factor-1 (IGF-1) from baseline.

**Figure 3.**
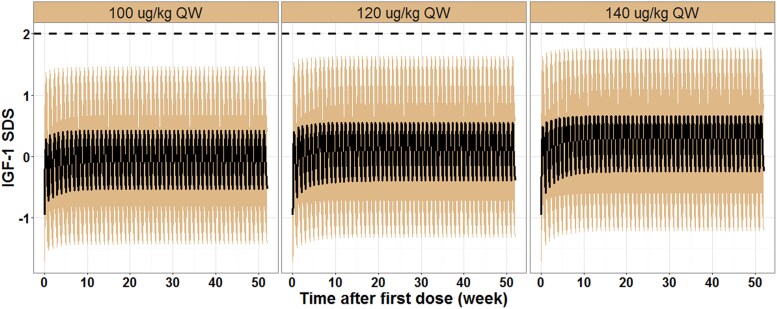
Quantitative pharmacology results: IGF-1 SDS profiles during 52 weeks after Pegpesen administration in children with GHD simulated by PK/PD model (n = 292). Abbreviations: GHD, growth hormone deficiency; IGF-1, insulin-like growth factor-1; PK/PD, pharmacokinetic/pharmacodynamic; SDS, SD score.

Analysis of the IGF-1 SDS curve with time data revealed that IGF-1 SDS levels in children with GHD increased slightly with escalating doses of Pegpesen, and levels following multiple doses were slightly higher than those after a single dose (Fig. 3). The IGF-1 SDS profile also indicated that the peak time for IGF-1 SDS after Pegpesen treatment ranged from 48 to 72 hours, with the time to reach average concentration ranging from 96 to 120 hours. Additionally, the maximum percentage of individuals with IGF-1 SDS peak concentration and average concentration greater than 2 was 3.08% (see Fig. 3), indicating that the current dosages of Pegpesen (100/120/140 μg/kg/week) were relatively safe.

### Safety

During the treatment period, the incidence of treatment-emergent adverse events (TEAEs) was 87.7% in the Pegpesen group and 94.6% in the daily rhGH group, with severity predominantly categorized as grade 1 and 2. The incidence of TEAEs related to trial medication was similar between the two groups, at 19.9% and 20.0%, respectively. TEAEs leading to temporary discontinuation of treatment occurred in 10.0% of the Pegpesen group and 27.7% of the daily rhGH group. The most common TEAEs leading to temporary discontinuation were fever (0.8% vs 11.5%) and upper respiratory tract infection (3.8% vs 6.9%). Notably, all fevers and upper respiratory tract infection resulting in temporary discontinuation were unrelated to the trial drugs and resolved spontaneously. No TEAEs resulting in dose reduction occurred in the Pegpesen group. No TEAEs resulting in death were observed during the trial. The incidence of serious adverse events (SAEs) was similar between the two groups, at 6.5% in the Pegpesen group and 6.2% in the daily rhGH group (Table 4).

**Table 4.** Adverse events (safety analysis set)

Item	Pegpesen group (N = 261)		Daily rhGH group (N = 130)	
	No. of cases (%)	No. of events	No. of cases (%)	No. of events
All TEAEs	229 (87.7)	1146	123 (94.6)	651
TEAEs related to trial drugs	52 (19.9)	112	26 (20.0)	51
TEAEs leading to dose reduction	0	0	1 (0.8)	2
TEAEs leading to increased dose	0	0	0	0
TEAEs leading to temporary discontinuation	26 (10.0)	41	36 (27.7)	68
TEAEs leading to permanent discontinuation	4 (1.5)	4	2 (1.5)	2
TEAEs leading to withdrawal from trial	4 (1.5)	4	2 (1.5)	2
TEAEs leading to death	0	0	0	0
All SAEs	17 (6.5)	26	8 (6.2)	11
SAEs related to trial drugs	1 (0.4)	1	0	0

Abbreviations: rhGH, recombinant human growth hormone; SAEs, serious adverse events; TEAEs, treatment-emergent adverse events.

TEAEs with an incidence of 5% or greater in the Pegpesen group mainly included upper respiratory tract infection (49.4% vs 44.6%), fever (18.4% vs 25.4%), cough (14.6% vs 19.2%), and bronchitis (10.3% vs 14.6%), followed by tonsillitis (7.3% vs 3.8%), indigestion (5.7% vs 4.6%), rhinorrhea (5.0% vs 3.8%), and allergic rhinitis (5.0% vs 3.8%). These were in line with those observed in the rhGH group. Additionally, 99% of these cases were deemed unrelated to the trial drugs. Other TEAEs related to GH, such as hypothyroidism, injection site pain, adrenal insufficiency, scoliosis, increased intracranial pressure, glucose metabolism abnormalities, dyslipidemia, and fluid retention, occurred very rarely in the Pegpesen group (0-2 cases each).

#### Immunogenicity results

Following 52 weeks of treatment, the rates of new antidrug antibodies and new neutralizing antibodies for Pegpesen were significantly lower than those observed in the daily rhGH group (0.0% vs 26.4%; *P* < .0001; 0.0% vs 7.9%; *P* < .0001).

### Adherence

The doses administered compared to the doses prescribed for the Pegpesen group and the daily rhGH group were 99.5% and 97.7%, respectively, indicating good adherence in both groups.

## Discussion

Currently, rhGH replacement therapy is primarily used in children with GHD to attain normal adult height (24). However, the daily regimen of rhGH treatment may lead to missed doses and nonadherence, resulting in inadequate growth responses (16). Since 1999, various LAGH formulations have been developed, some of which have been approved for clinical use, aiming to improve pediatric compliance and, consequently, therapeutic outcomes and quality of life (11, 25). LAGH formulations approved by the US Food and Drug Administration and European Medicines Agency for use in children include somatrogon (Ngenla), lonapegsomatropin (Skytrofa) and somapacitan (Sogroya) (16), while China has authorized the use of only one LAGH product (Jintrolong) (3). The mentioned LAGHs employ different long-acting technologies, with both Jintrolong and Pegpesen using PEGylation, though the PEG structures and modification sites differ between the two (18). This might result in variations in biological activity, half-life, and recommended dosage (Jintrolong 200 μg/kg/week vs Pegpesen 140 μg/kg/week) (18).

In terms of drug technology, Pegpesen is modified with a novel Y-shaped PEG chain, which provides superior protective effects on rhGH and relatively higher protein biological activity. The previous phase 2 trial suggested that Pegpesen is suitable for the once-weekly treatment of pediatric GHD (18). This phase 3 study found that Pegpesen was noninferior to daily rhGH in terms of GV, with a similar safety profile. It is worth noting that the dosage of Pegpesen and daily rhGH was 140 μg/kg/week and 245 μg/kg/week, respectively. Therefore, while offering comparable efficacy and safety, the dosage of Pegpesen is merely 57% of the equivalent dose of daily rhGH and is also lower than other approved LAGH formulations (10, 17). Considering the nature of GH drugs, GV is closely linked to drug dosage; however, higher-dosage GH regimens and growth rates may pose additional safety concerns (26). Hence, administering Pegpesen at a lower dose to children requiring long-term treatment may result in better long-term safety outcomes. This approach aligns with the goal of optimizing therapeutic strategies while minimizing potential risks associated with treatment.

Recently, a real-world study showed that adherent patients gained an additional 1.8 cm in height during the first year of therapy compared to nonadherent patients (27). In multiple phase 3 studies of LAGH with a treatment duration of 52 weeks, the average adherence to medical orders ranged from 95.8% to 99.6%, which is similar to the adherence of the Pegpesen group (99.3%) in this study (19-21). Despite no statistically significant difference in the adherence between the Pegpesen group and the daily rhGH group in this study, the reduction in injection frequency helps to alleviate the treatment burden for children with GHD and their caregivers. Furthermore, in the future it will be necessary to observe the gap in compliance between Pegpesen and daily rhGH, as well as the resulting differences in therapeutic efficacy over a longer treatment period in the real world.

Immunogenicity is a substantial concern for LAGH drugs, as the additional molecular and pharmaceutical manipulations involved in their production may lead to the formation of neutralizing antibodies and increased immunogenicity, thereby reducing drug bioavailability and efficacy (28). In the present study, the positive rates of new antidrug antibodies and new neutralizing antibodies were both 0.0% in the Pegpesen group, significantly lower than those in the rhGH group (*P* < .0001). In 2 other phase 3 studies of LAGHs, Skytrofa and Soroya, the positive rate of antidrug antibodies at 52 weeks was comparable to that of daily rhGH (6.7% vs 3.6%, 1.5% vs 1.5%) (19, 20). Moreover, in a phase 3 study focusing on the once-weekly GH-fusion protein somatrogon, 15.6% of participants tested positive for antidrug antibodies (21). Additionally, in the phase 3 study of UPEG-rhGH, no antidrug antibodies were generated during the 26-week treatment period (17). This study is the first to demonstrate that PEGylation rhGH does not induce the production of antidrug antibodies during a treatment period of up to 52 weeks, indicating that Pegpesen can effectively mitigate immunogenicity, aligning with the initial design objectives of PEG-modified drugs (12).

Regarding safety, the overall incidence of TEAEs in the Pegpesen group was similar to that in the rhGH group, with severity predominantly categorized as grade 1 and 2, often alleviated spontaneously or with simple treatment. Furthermore, the incidence of medication discontinuation due to TEAEs was lower in the Pegpesen group compared to the rhGH group (10% vs 27.7%). The improved medication continuity in the Pegpesen group may be attributed to higher full adherence to medical orders and could partly explain why the Pegpesen dosage is only 57% of the equivalent dose of rhGH to achieve similar therapeutic effects. It is a concern that LAGH may potentially cause metabolic abnormalities (28). However, in this study, Pegpesen hardly affected the normal glucose metabolism, lipid metabolism, and thyroid function of the participants.

The growth effect of GH is primarily attributed to its ability to induce IGF-I. Serum IGF-I concentration is commonly used as a biomarker to assess the efficacy and safety of rhGH medication (24, 29). Therefore, research on each LAGH should determine the optimal time for IGF-1 assessment, aiming to maintain serum IGF-1 concentrations within the age-appropriate normal range (–2SD to 2SD) for most of the treatment period (25). This study established a quantitative pharmacological model based on PK/PD data from phase 2/3 participants. The data revealed that the IGF-1 SDS peak time after Pegpesen 100/120/140 μg/kg/week treatment ranged from 48 to 72 hours, with time to reach the average concentration being 96 to 120 hours, guiding the timing of regular monitoring in clinical practice. In the present study, the maximum percentage of individuals with IGF-1 SDS peak concentration and average concentration greater than 2 was 3.08%, slightly lower than Jintrolong (7.5%) (17) and Skytrofa (3.8%) (19), indicating the relatively safe IGF-1 SDS range following Pegpesen 100 to 140 μg/kg/week treatment. Additionally, at 12 weeks, the ΔIGF-1 AUC of each Pegpesen dose group had reached a steady state, similar to the ΔIGF-1 AUC at 52 weeks. Thus, the ΔIGF-1 AUC at 12 weeks can determine the Pegpesen dosage equivalent in efficacy and safety to daily rhGH. Therefore, from the perspective of safety, combined with the quantitative pharmacological model established with IGF-1 and the clinical efficacy measured by GV, the dose selection in this study is reasonable.

It is worth noting that the cutoff values for the GH stimulation test used to diagnose GHD vary across countries. In the United States and China, the cutoff value is 10 μg/L, whereas in Italy and Germany it is 8 μg/L, and in France and the United Kingdom it is 6.7 μg/L (30, 31). Therefore, the diagnostic criteria for GHD remain controversial, especially for patients with peak GH levels between 7 and 10 μg/L. This study conducted a subgroup analysis based on different GH peak levels (≤5 μg/L, <5 μg/L to ≤7 μg/L, and >7 μg/L) and found that in each subgroup, the LS means of GV in the Pegpesen group was not inferior to that of the daily rhGH group, with similar results observed in both groups (data not shown). Among these, the LS means of GV for participants with peak GH levels between 7 and 10 μg/L was similar to the main analysis, indicating significant benefits for this subgroup as well. However, the standards for stimulation tests in the diagnosis of GHD remain a scientific issue that requires further exploration and more research in the future to establish standardized criteria.

This study had some limitations. First, it employed an open-label design due to the different administration frequencies of drugs in the two groups, potentially introducing slight bias into the results. Second, the treatment and observation duration in this study was approximately 1 year, warranting longer follow-up periods to ascertain the long-term safety, efficacy, and adherence of Pegpesen.

Future research directions could involve evaluating the treatment burden on patients (including financial, physiological, and psychological aspects), as well as assessing the efficacy and safety of long-term treatment. Additionally, exploring the feasibility of extending dosing intervals to further reduce dosing frequency for patients would be valuable. Moreover, investigating the clinical application of Pegpesen in a broader population of individuals with GHD would benefit more patients.

## Data Availability

Some or all data sets generated during and/or analyzed during this study are not publicly available but are available from the corresponding author on reasonable request.

## Clinical Trial Information

ClinicalTrials.gov clinical trial registration number: NCT04513171 (registered <2020-08-07>).

## Abbreviations

AUC: area under the curve
BA: bone age
CA: chronological age
GH: growth hormone
GHD: growth hormone deficiency
GV: growth velocity
Ht: height
IGF-1: insulin-like growth factor-1
IGFBP-3: insulin-like growth factor-binding protein 3
LAGH: long-acting growth hormone
LS means: least squares mean
PD: pharmacodynamic
PEG: polyethylene glycol
PK: pharmacokinetic
rhGH: recombinant human growth hormone
SAEs: serious adverse events
SDS: SD score
TEAEs: treatment-emergent adverse events
UPEG-rhGH: U-shaped branched *N*-terminal PEGylated rhGH
